# Comprehensive Analysis of TICRR in Hepatocellular Carcinoma Based on Bioinformatics Analysis

**DOI:** 10.1007/s10528-023-10378-w

**Published:** 2023-06-02

**Authors:** Jing-Jing Chen, Lu-Lu Zhang, Zhen Liu, Wan Qi Men, Fang Chen, Jilu Shen

**Affiliations:** 1 Department of Clinical Laboratory, The First Affiliated Hospital of Anhui Medical University, Hefei, Anhui China; 2 Department of Clinical Laboratory, Anhui Public Health Clinical Center, Hefei, Anhui China; 3 Public Center of Experimental Technology, The School of Basic Medical, Science and Southwest Medical University, Luzhou, Sichuan China; 4 UItrasonic Diagnosis Deparment, The First Affiliated Hospital of Anhui Medical University, Hefei, Anhui China; 5 UItrasonic Diagnosis Deparment, Anhui Public Health Clinical Center, Hefei, Anhui China

**Keywords:** Hepatocellular carcinoma, HCC, Prognosis, Methylation, Immune infiltration, T-cell exhaustion

## Abstract

Hepatocellular carcinoma (HCC) is one of the leading cause of cancer-associated death in the world. However, due to the complexity of HCC, it is urgent for us to find a reliable and accurate biomarker for HCC gene therapy.TopBP1-interacting checkpoint and replication regulator (TICRR), known as Treslin in vertebrate and sld3 in yeast, is involved in the tumorigenesis, progression, matastasis, diagnosis, and predicting prognosis of HCC. Disappointingly, the mechanism of TICRR expression in HCC is still not described in detail and requires further analysis. In this study, TCGA (www.tcga-data.nci.nih.gov/tcga/) datasets and GEO (www.ncbi.nlm.nih.gov/geo) datasets were used to analyze the expression of TICRR in HCC, the relevance of TICRR mRNA expression and clinicopathological characteristics in patients with HCC, and the relationship between TICRR expression and immune infiltration level in Patients with HCC. Based on MethSurv database, the impact of TICRR in patients with HCC was investigated. In addition, GO/KEGG enrichment analysis of TICRR co-expression was performed using the R package. TICRR was found drastically highly expressed in a variety of cancer types including HCC.ROC curve analysis showed that TICRR had higher accuracy in predicting HCC compared with AFP. The expression level of TICRR was marked positively correlated with tumor stage and prognosis in Patients with HCC.GO/KEGG enrichment analysis showed that TICRR was associated with cell division and cell cycle as well as p53 signaling pathway. In addition, patients with high TICRR methylation of cg05841809, cg09403165, and cg03312532 CpG sites were significantly correlated with poor prognosis of HCC. This study demonstrated that increased TICRR expression in HCC might play an important role in the tumorigenesis, progression, diagnosis, and predicting prognosis of HCC. Therefore, TICRR might be used as a promising diagnostic and prognostic biomarker for HCC gene therapy.

**Supplementary Information:**

The online version contains supplementary material available at 10.1007/s10528-023-10378-w.

## Introduction

HCC is one of the most common malignant tumors in the world with high incidence rates and mortality rates (Chen et al. 2020; Meischl et al. 2021). However, 55 percent of the patients with HCC are Chinese people. In china, nowadays, the incidence of HCC is 28.71/10^5^ and the mortality rate is 26.04/10^5^ according to the related researches. In addition to that, the incidence and mortality rates of HCC have gradually increased year by year (Ho et al. 2022). The alpha-fetoprotein (AFP) is one of the most widely used HCC biomarkers in the world (Zhu et al. 2018a), but it is still controversial as a HCC biomarker (Yuan et al. 2019; Ding et al. 2020). Other HCC biomarkers, such as α-L-fucosidase (AFU), fucosylated fraction of AFP (AFP-L3) (Chalasani et al. 2021), carbohydrate antigen 199 (CA199), and carcinoembryonic antigen (CEA), have also been reported to have some limitations in the diagnosis of HCC. Therefore, it is critical for us to search for a more convincing molecular biomarker and therapeutic target for the early diagnosis and comprehensive monitoring of HCC.

TopBP1-interacting checkpoint and replication regulator (TICRR) is the regulator of DNA replication and S/M and G2/M checkpoints (Yang et al. 2021; Wittig et al. 2021). TICRR regulates the triggering of DNA replication initiation via its interaction with TOPBP1 by participating in CDK2-mediated loading of CDC45L onto replication origins. TICRR, a critical DNA replication initiation regulator, is required for the transition from pre-replication complex (pre-RC) to pre-initiation complex (pre- IC). Currently, some studies have already demonstrated that TICRR is overexpressed and involved in tumor carcinogenesis, progression, and chemotherapeutic drug resistance process (Yang et al. 2021; Wang et al. 2021). An increasing number of studies have demonstrated that TICRR pathway plays a critical role in the progression of numerous solid tumors, such as head and neck squamous cell carcinoma, colon adenocarcinoma, liver hepatocellular carcinoma, cholangiocarcinoma, gastric adenocarcinoma, colorectal cancer, prostate cancer, rectal adenocarcinoma, lung adenocarcinoma, lung squamous cell carcinoma, breast invasive carcinoma, esophageal carcinoma, kidney renal clear cell carcinoma, endometrial cancer, kidney renal papillary cell carcinoma, and so on. However, our understanding of the main molecular mechanism and predominant signaling pathways of TICRR in tumorigenesis and progression of HCC is still limited.

In this study, we comprehensively analyzed the differences of TICRR expression in diverse cancer types including HCC, clinicopathological relevance, diagnostic and prognostic value, DNA methylation level, and the underlying functional mechanisms of TICRR gene in HCC using The Cancer Genome Atlas (TCGA), Kaplan–Meier Plotter, TIMER 2.0, GEO, and various public databases. Immune microenvironment has been recognized as an important factor in the process and development of cancer. The metastatic potential of cancer is influenced by the local immune microenvironment which may play significant roles in promoting tumorigenesis and progression. Therefore, the correlations between TICRR expression and immune Infiltration, immune cell biomarkers, and immune checkpoints were also investigated. Besides, gene functional networks and protein–protein interaction (PPI) analysis were performed to explore protein interaction of TICRR gene in HCC. The purpose of this study is to provide more data for discovering the potential biological mechanisms of TICRR gene which could be helpful for the research of immunotherapy for HCC.

## Materials and Methods

### TCGA Data

TCGA database is an online analysis website. It contains more than 10, 000 samples, which can be divided into 39 tumor types. HCC RNA-seq data were downloaded from the Genomic Data Commons (GDC, https://portal.gdc.cancer.gov/) database, including 374 liver tumor samples and 50 normal liver samples.

### GEO Data

In order to analyze the difference of TICRR transcription level between HCC tissues and normal liver tissues, we downloaded the RNA sequencing data of HCC from the Gene Expression Omnibus (GEO) database (GSE102079, *n* = 508).

### RNA Extraction and qRT-PCR

All liver tissue samples were obtained from patients with LHC who underwent radical resection at the First Affiliated Hospital of Anhui Medical University from September 2022 to November 2022. The total RNA was extracted from liver tissue samples using TRIzol (Invitrogen) following the manufacturer’s instructions. Reverse transcription was performed using an RT Kit (TakaRa, Dalian, China) in accordance with the manufacturer’s protocol. Quantitative real-time PCR was performed on a Light Cycler96 Detection System using SYBR Green RT PCR kit (Thermo Scientific). In addition, GAPDH was used as an internal reference and the 2 −△△Ct method was used to calculate the results. TICRR forward primer sequences are CTTTGTGGCCTTCTTTGAAGT and reverse primer sequences are CCACACAGTTGCTCCACAT. The forward primer sequences and reverse primer sequences of GAPDH were GGAGCGAGATCCCTCCAAAAT and GGCTGTTGTCATACTTCTCATGG, respectively.

### STRINGS Analysis

All protein–protein interaction (PPI) data were obtained from STRINGS (www.string-db.org) database. In this study, the STRING database was applied to comprehensively analyze the Protein–Protein Interaction of TICRR gene in HCC.

### MethSurv Analysis

METHSURV (https://biit.cs.ut.ee/MethSurv/), a web-based tool for univariate and multivariate survival analyses based on DNA methylation biomarkers using TCGA data, contains 25 different types of cancer and 7358 patients.

### R Software

Data were analyzed using R (version 3.6.3) (statistical analysis and visualization), R Package: survminer package [version 0. 4.9] (for visualization), and survival package [version 3.2–10] (for statistical analysis of survival data). GO/KEGG enrichment analysis of TICRR co-expression was performed using the ClusterProfiler package (version 3.18.0). The ggplot2 software package was used to analyze the data. Tumors were classified into high and low TICRR expression groups according to the median normalized to Z score by Ggplot2 [version 3.3.3] (for visualization) and using the main parameters: LogFC > 2 and *P* value < 0.01 as the threshold for statistical difference. The results of the differential analysis are shown using volcano and heat maps.

### Statistical Analysis

Differences between groups were compared using Wilcoxon test (TIMER2.0) or Student’s t test (Oncomine and UALCAN). The effect of the treatment on each parameter was analyzed by one-way analysis of variance (ANOVA). Results are shown by mean ± SD, and difference was statistically significant when *P* value is less than 0.05.

## Results

### Pan-Cancer Analysis of TICRR mRNA Expression

**Fig. 1:**
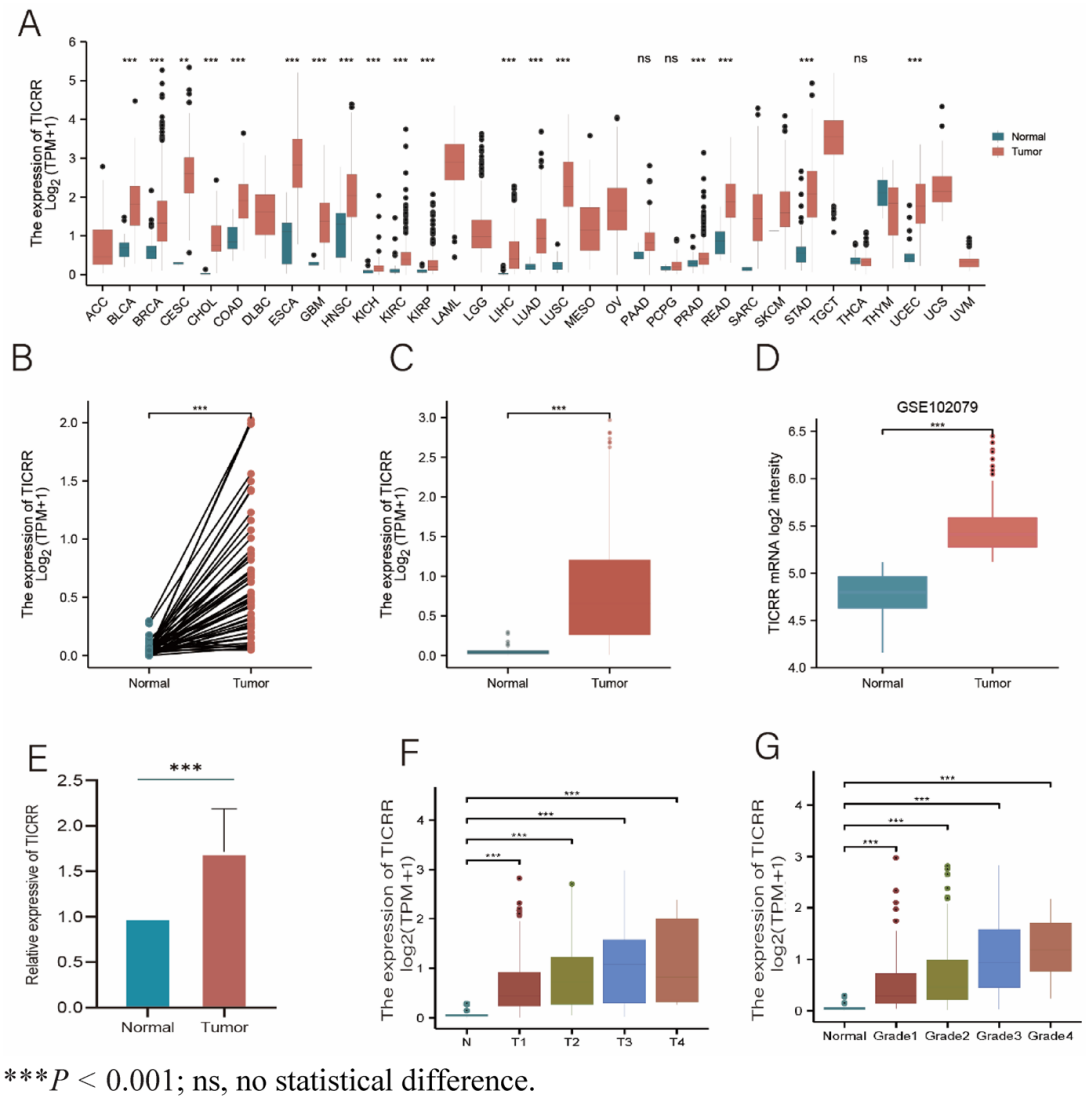
The comparison of TICRR expression between cancer and normal tissues. **A** The expression of TICRR in Various Tumors was higher than that in normal tissues in TCGA database. **B** Compared with unmatched 50 normal liver tissues, TICRR was significantly upregulated in 374 HCC tissues. **C** Compared with paired 50 normal liver tissues, TICRR was significantly upregulated in 50 HCC tissues. **D** TICRR expression was higher in HCC than that in normal liver tissues in GSE102079. **E** QRT-PCR showed that the expression of TICRR mRNA in liver tumor tissues was significantly higher than that in paired normal liver tissues. **F** High expression of TICRR was significantly correlated with T stage. **G** High expression of TICRR was significantly correlated with histological grade. ***P < 0.001; ns, no statistical difference

Based on the TCGA database, the differences in TICRR expression in various tumors and normal tissues were analyzed. The results showed that TICRR was radically highly expressed in different types of cancers compared with normal tissues (Fig. 1A). Compared with normal tissues, TICRR was significantly higher in BLCA (bladder urothelial carcinoma), ESCA (esophageal carcinoma), HNSC (head and neck squamous cell carcinoma), COAD (colon adenocarcinoma), LIHC (liver hepatocellular carcinoma), LUAD (lung adenocarcinoma), lUSC (lung squamous carcinoma), THCA (thyroid adenocarcinoma), STAD (gastric adenocarcinoma), READ (rectal adenocarcinoma), and UCEC (endometrial cancer). However, the expression of TICRR was remarkably lower in KICH (Kidney Chromophobe), KIRC (Kidney renal clear cell carcinoma), and KIRP (Kidney renal papillary cell carcinoma).

### Transcriptional Levels of TICRR in Patients with HCC

In order to obtain the transcriptional Levels of TICRR in patients with HCC, we used TCGA datasets and GEO datasets for analysis. There were datasets showing that TICRR mRNA expression was dramatically upregulated in HCC compared with that in unpaired and paired normal liver tissues (Fig. 1B, C, D). To validate the accuracy of the data analysis, we also used qRT-PCR to detect TICRR mRNA expression in liver tumor tissues. The results of qRT-PCR showed that the expression of TICRR mRNA in liver tumor tissues was remarkably higher than that in paired normal liver tissues (Fig. 1E). These results may indicate that TICRR probably has a potential carcinogenic effect on the development and progression of HCC.

### Correlation Analysis of TICRR Expression and Clinicopathological Parameters in HCC

Based on TCGA database, we investigated the relevance of TICRR Expression and Clinicopathological features in Patients with HCC. The results showed that the high expression of TICRR was noticeably correlated with age, T stage, Pathologic stage, histological grade, AFP concentration, OS event, and DSS event (*P* < 0.05). However, no significant difference was found for TICRR expression in HCC patients of different genders and Albumin concentration (Table 1; Fig. 1F, G).

**Table 1:** Relationship between TICRR mRNA expression and clinicopathological characteristics in patients with HCC

Characteristic	Low expression of TICRR	High expression of TICRR	*p*
*n*	187	187	
Age, meidan (IQR)	64 (54, 69)	59 (51, 67)	0.013*
Gender, *n* (%)			0.077
Female	52 (13.9%)	69 (18.4%)	
Male	135 (36.1%)	118 (31.6%)	
T stage, *n* (%)			0.004*
T1	108 (29.1%)	75 (20.2%)	
T2	40 (10.8%)	55 (14.8%)	
T3	30 (8.1%)	50 (13.5%)	
T4	6 (1.6%)	7 (1.9%)	
Pathologic stage, n (%)			0.002*
Stage I	101 (28.9%)	72 (20.6%)	
Stage II	38 (10.9%)	49 (14%)	
Stage III	31 (8.9%)	54 (15.4%)	
Stage IV	4 (1.1%)	1 (0.3%)	
Histologic grade, *n* (%)			< 0.001*
G1	41 (11.1%)	14 (3.8%)	
G2	101 (27.4%)	77 (20.9%)	
G3	42 (11.4%)	82 (22.2%)	
G4	1 (0.3%)	11 (3%)	
AFP (ng/ml), *n* (%)			< 0.001*
≤ 400	133 (47.5%)	82 (29.3%)	
> 400	13 (4.6%)	52 (18.6%)	
Albumin (g/dl), *n* (%)			0.444
< 3.5	41 (13.7%)	28 (9.3%)	
≥ 3.5	123 (41%)	108 (36%)	
OS event, *n* (%)			0.023*
Alive	133 (35.6%)	111 (29.7%)	
Dead	54 (14.4%)	76 (20.3%)	
DSS event, *n* (%)			0.022*
Alive	153 (41.8%)	134 (36.6%)	
Dead	30 (8.2%)	49 (13.4%)	

* and bold values indicate *P* < 0.05

### The Diagnostic and Predictive Value of TICRR in HCC Patients

**Fig. 2:**
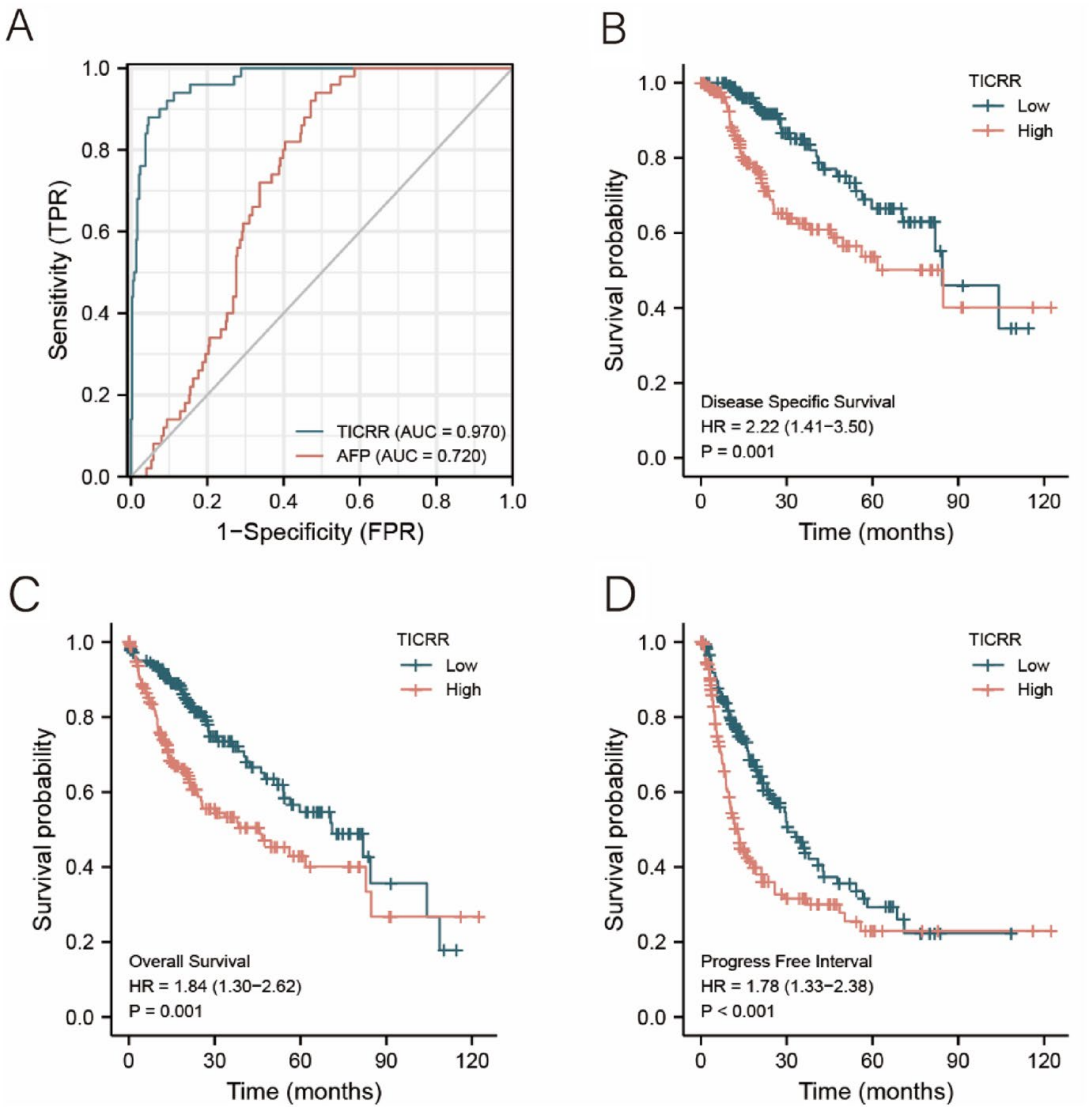
The diagnostic and predictive value of TICRR in HCC Patients. **A** ROC curve analysis showed the diagnostic value of TICRR and AFP in HCC Patients. **B** Disease-specific survival, (**C**) overall survival, and (**D**) progression-free survival

To further evaluate the diagnostic value of TICRR in HCC Patients, we used a ROC curve to describe it. It is well known that AFP is a commonly used classical marker in predicting HCC. ROC curve analysis showed that the area under the curve (AUC) of TICRR and AFP was 0.970 (CI 0.951–0.988) and 0.720 (CI 0.668–0.773), respectively (Fig. 2A). This suggests that TICRR was more sensitive and specific than AFP for HCC diagnosis. Moreover, as shown in Figs. 2B–D, overall survival (HR 1.84, *P* < 0.001), progression-free survival (HR 1.78, *P* < 0.001), and disease-specific survival (HR 2.22, *P* < 0.001) in high expression of TICRR groups were all statistically worse than those in the low expression of TICRR groups. These results may indicate that increased TICRR expression is associated with poor prognosis of HCC.

### Enrichment Analysis of TICRR Gene Functional Networks and PPI Network in HCC

**Fig. 3:**
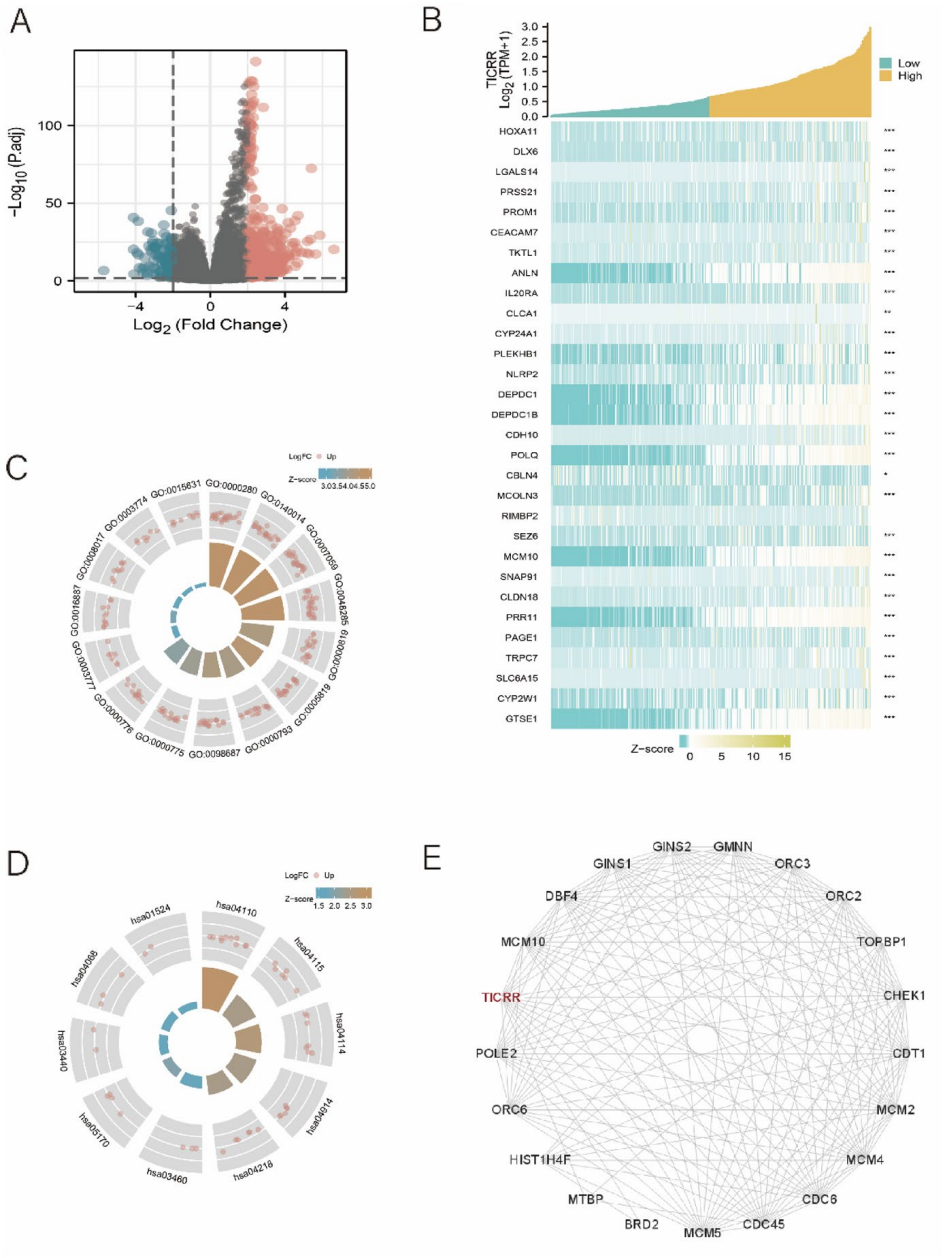
Enrichment analysis of TICRR Gene functional networks in HCC. **A** Genes significantly correlated with TICRR in HCC **B** The top 30 genes positively correlated with TICRR Gene in HCC **C** GO enrichment analysis of TICRR **D** KEGG pathway enrichment analysis of TICRR **E** Protein interaction network of TICRR in HCC

The biological function of TICRR in HCC was further investigated by enrichment analysis of TICRR Gene functional networks and PPI Network. Enrichment analysis showed that 755 genes were positively correlated with TICRR gene and 116 genes were negatively correlated with TICRR gene (LogFC > 2 and *P* value < 0.01) (Fig. 3A). Moreover, the top 30 genes that were significantly positively correlated with TICRR gene are shown in Fig. 3B. In addition to that, R software package was used to perform Gene ontology (GO) and Kyoto Encyclopedia of Genes and Genomes (KEGG) enrichment analysis of TICRR-related genes. Under the condition of p.adj < 0.1, there are 84 biological processes (GO-BP), 8 cellular components (GO-CC), 5 biological processes (GO-MF), and 1 KEGG. The bubble chart showed the first 15 pieces of information about GO and KEGG, including 5 pieces of BP, CC, and MF. GO enrichment analysis showed that TICRR was associated with cell division (Fig. 3C). KEGG pathway enrichment analysis of TICRR showed that TICRR co-expression is mainly related to cell cycle and p53 signaling pathway (Fig. 3D, Appendix 1). To further understand protein interaction of TICRR gene in HCC, we constructed a PPI network of TICRR gene (Fig. 3E). The analysis showed that Topoisomerase (dna) II-binding protein 1 (TOPBP1), Cell division control protein 45homolog (CDC45), Mdm2-binding protein (MTBP), Minichromosome maintenance complex component 2 (MCM2), Protein MCM10 homolog (MCM10), Protein DBF4 homolog (DBF4), Serine/threonine-protein kinase Chk1 (CHEK1), DNA replication factor Cdt1 (CDT1), Cell division control protein 6 homolog (CDC6), and Bromodomain-containing protein 2 (BRD2) were recognized as the top ten correlated genes in the PPI network. The interaction scores were 0.997, 0.996, 0.985, 0.958, 0.933, 0.901, 0.903, 0.909, 0.894, and 0.891, respectively. TOPBP1 and CDC45 play an important role in DNA replication, and MTBP plays a critical role in promoting the growth and migration of tumor cells.

The results showed that TOPBP1, CDC45, and MTBP had the highest correlation with TICRR in HCC. These results may indicate that TICRR might play an important role in the tumorigenesis and progression of HCC.

### Analysis of DNA Methylation Level of TICRR in Patients with HCC

**Fig. 4:**
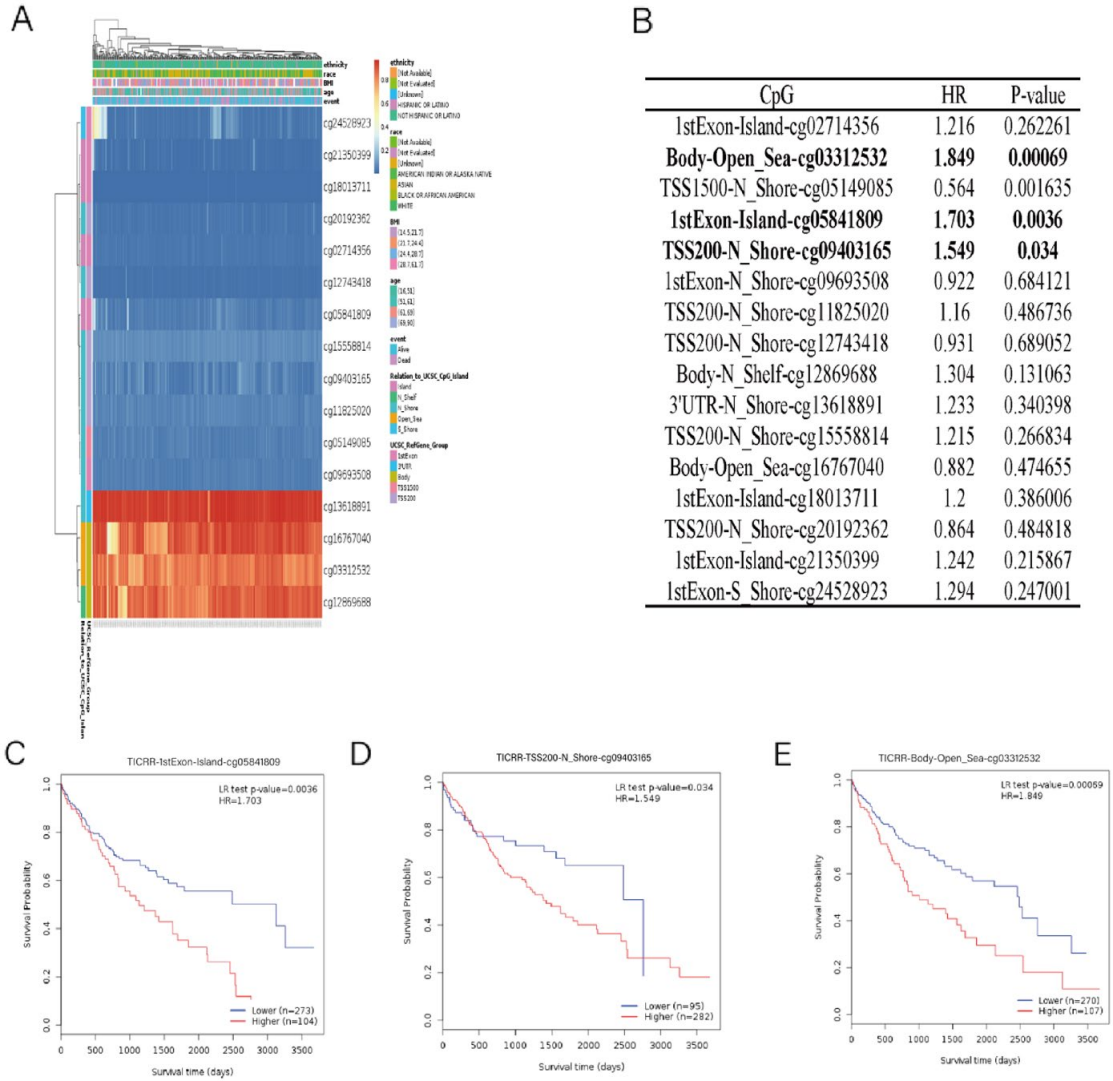
Relationship between TICRR expression and the methylation level in patients with HCC. **A** Associations between TICRR expression and the methylation level. **B** Effect of high TICRR methylation level of different CpG sites on prognostic value in HCC. **C** OS of patients with different TICRR methylation levels of cg05841809 CpG sites. **D** OS of patients with different TICRR methylation levels of cg09403165 CpG sites. **E** OS of patients with different TICRR methylation levels of cg03312532 CpG sites

we used MethSurv (https://biit.cs.ut.ee/methsurv/) to further evaluate the correlations between methylation levels of cytosine-phosphate-guanine (CpG) sites of TICRR and OS of HCC patients. MethSurv analysis showed 16 methylated CpG sites, of which cg13618891 CpG site had the highest degree of DNA methylation level of TICRR (Fig. 4A). Moreover, as shown in Fig. 4B, high TICRR methylation levels of cg05841809, cg09403165, and cg03312532 were dramatically correlated with the prognostic values in HCC (*P* < 0.05). In order to study the relationship between methylation levels of cytosine-phosphate-guanine (CpG) sites of TICRR and OS of HCC patients, methylation level and survival time were used as explanatory variable and response variable, respectively. Kaplan–Meier diagram showed that patients with high TICRR methylation of cg05841809, cg09403165, and cg03312532 CpG sites had a worse overall survival (OS) than patients with low TICRR methylation (Fig. 4C–E).

### Correlation Analysis Between TICRR Expression and Immune Infiltration in HCC

**Fig. 5:**
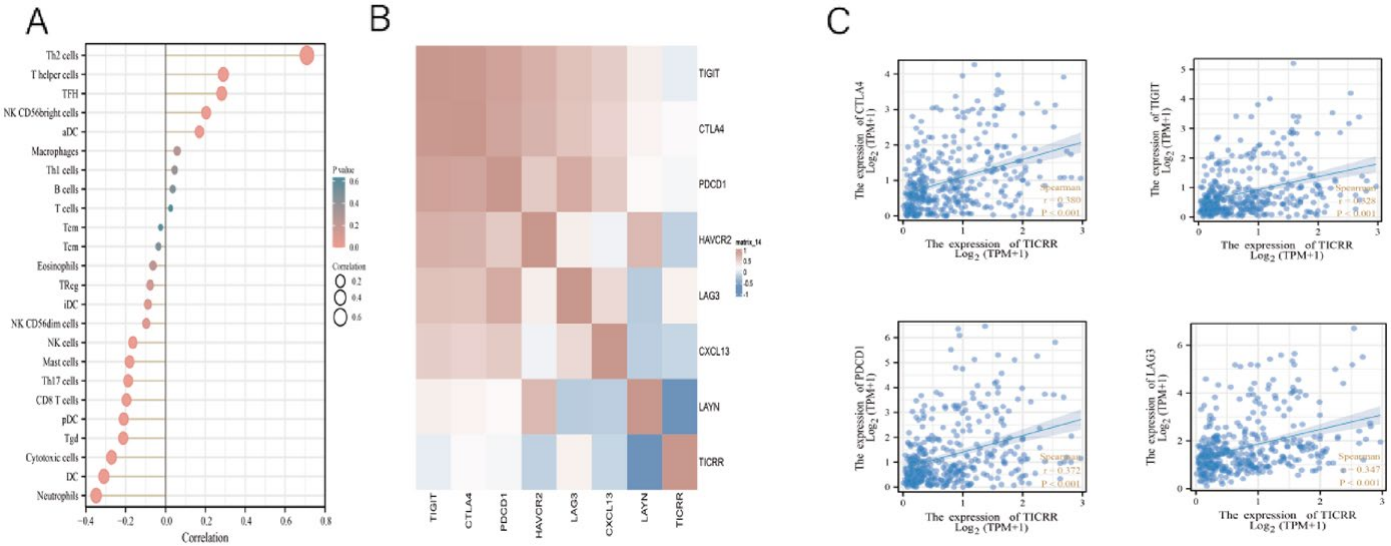
Correlation between high expression of TICRR and immune infiltration in HCC. **A** TICRR expression was significantly correlated with infiltrating levels of B-cells, CD8 + T-cells, T-cells, CD4 + T-cells, macrophages, neutrophils, and DCs. **B** Heat map of correlation between TICRR expression and PDCD1, CTLA4, HAVCR2, LAG3, TIGIT, LAYN, and CXCL13. **C** The scatter plot of correlation between TICRR expression and PDCD1, CTLA4, LAG3, and TIGIT

In order to study the relationship between TICRR expression and biomarkers of immune cells in HCC, the TIMER database was used to describe it. The analysis showed that the expression level of TICRR was noticeably correlated with B-cell biomarkers (CD19 and CD79A), CD8 + T-cell biomarkers (CD8A and CD8B), T-cell biomarkers (CD3D, CD3E, and CD2), other T-cell subsets (Th1 and Th2), Monocyte biomarkers (CD86 and CSF1R), TAM biomarkers (CD68 and IL10), M1 macrophage biomarkers (IRF5), neutrophil biomarkers (ITGAM and CCR7), natural killer cell biomarkers ( B3GAT1 and CD7), dendritic cell biomarkers (CD1C), and exhausted T-cells biomarkers (PDCD1, HAVCR2, TOX, NRP1, LAG3, SLAMF6, and TIGIT) in HCC (*P* < 0.05, Table 2). In conclusion, the expression level of TICRR was significantly positively correlated with infiltrating levels of CD8 + T-cells (*r* = − 0.187, *p* = 0.0003), T helper cells (*r* = 0.313, *p* = 6.3821E-10), Cytotoxic cells (*r* = − 0.262, *p* = 2.7209E-07) and neutrophils (*r* = − 0.344, *p* = 8.009E-12) in HCC (Fig. 5A). Moreover, exhausted T-cells have been found in a variety of tumors. PDCD1, CTLA4, LAG3, and TIGIT are important markers in exhausted T-cells.They are also predictive markers of therapeutic effect of immune checkpoint inhibitors (ICIS). The results showed that the expression of PDCD1, CTLA4, LAG3, and TIGIT are positively correlated with TICRR in HCC (*r* = 0.372, *r* = 0.380, *r* = 0.347, *r* = 0.328, *P* < 0.001) (Fig. 5B, C).

**Table 2:** Correlation between TICRR expression and biomarkers of immune cells in HCC

Description	Gene markers	TIMER	
		cor	p
B-cell	CD19	0.293565	8.27E-09*
	CD79A	0.228241	8.99E-06*
CD8 + T-cell	CD8A	0.233898	5.62E-06*
	CD8B	0.217803	2.32E-05*
T-cell	CD3D	0.308213	1.61E-09*
	CD3E	0.240159	3.09E-06*
	CD2	0.255278	6.84E-07*
Monocyte	CD86	0.320384	3.36E-10*
	CSF1R	0.171668	0.000914*
TAM	CCL2	0.091294	0.079063
	CD68	0.246777	1.62E-06*
	IL10	0.256914	5.27E-07*
M1 Macrophage	NOS2	-0.00552	0.915602
	IRF5	0.418484	3.67E-17*
M2 Macrophage	CD163	0.091709	0.077697
	VSIG4	0.089812	0.084069
	MS4A4A	0.09094	0.080237
Neutrophil	CEACAM8	0.100493	0.053114
	ITGAM	0.312983	8.79E-10*
	CCR7	0.125546	0.015537*
Natural killer cell	KIR2DL1	-0.02077	0.690087
	B3GAT1	0.167178	0.001229*
	CD7	0.334099	5.25E-11*
Dendritic cell	CD1C	0.158915	0.00214*
	THBD	-0.05074	0.329711
Th1	IL12RB1	0.292217	1.14E-08*
	CCR1	0.250112	1.16E-06*
	CCR5	0.299703	3.88E-09*
Th2	GATA3	0.262041	3.06E-07*
	STAT6	0.10223	0.049115*
	STAT5A	0.323005	1.86E-10*
	IL13	0.126269	0.014947*
Tfh	BCL6	0.170769	0.000973*
	IL21	0.146229	0.004769*
Th17	IL23R	0.236546	4.10E-06*
	CCR6	0.368409	2.27E-13*
Treg	FOXP3	0.165139	0.001413*
	NT5E	0.122206	0.018585*
	IL7R	0.137082	0.008194*
Exhausted T-cells	PDCD1	0.361585	6.69E-13*
	HAVCR2	0.337687	3.18E-11*
	TOX	0.219681	1.96E-05*
	NRP1	0.254873	**7.13E-07**
	LAG3	0.346095	9.53E-12*
	SLAMF6	0.177554	0.000591*
	TIGIT	0.334422	3.82E-11*

* and bold values indicate *P* < 0.05

## Discussion

Hepatocellular carcinoma (HCC) is a common malignancy around the world which has seriously threatened and damaged human’s health (Wang et al. 2020; Zhang and Zhang 2019). In recent years, gene therapy has become the most potential treatment for numerous cancers including HCC in biomedical field, following the development in recognition of molecular process of diseases and technologies in molecular biological field (Dalwadi et al. 2021; Kamimura et al. 2020; Dong et al. 2020). However, the main molecular biological mechanism of TICRR in tumorigenesis, occurrence, metastasis, and recurrence of HCC is still unclear. Therefore, investigating the gene expression mechanisms of HCC is the key for HCC gene therapy.

In our research, we found that TICRR was dramatically highly expressed in a variety of cancer types including HCC, compared with normal tissues based on TCGA database. In addition, TICRR mRNA expression was noticeably upregulated in HCC compared with that in unpaired and paired normal liver tissues. To validate the accuracy of the data analysis, we also used qRT-PCR to detect TICRR mRNA expression in liver tumor tissues. The results of qRT-PCR showed that the expression of TICRR mRNA in liver tumor tissues was remarkably higher than that in paired normal liver tissues. These results may indicate that TICRR probably has a potential carcinogenic effect on the development and progression of HCC. Furthermore, the high expression of TICRR was remarkably correlated with age, T stage, Pathologic stage, Histologic grade, AFP concentration, OS event, and DSS event. ROC curve analysis showed that the area under the curve (AUC) of TICRR and AFP was 0.970 (CI 0.951–0.988) and 0.720 (CI 0.668–0.773), respectively**.** This suggests that TICRR had the higher diagnostic value than AFP in HCC. Moreover, overall survival (HR 1.95, *P* < 0.001), progression-free survival (HR 1.88, *P* < 0.001), and disease-free survival (HR 2.46, *P* < 0.001) in high expression of TICRR groups were all statistically worse than those in the low expression of TICRR groups. Therefore, TICRR can be used as a precise diagnostic and prognostic molecular biomarker for gene therapeutic strategies of HCC.

Through the TICRR Gene co-expression networks and PPI network analysis of TICRR in HCC, it was found that the Topoisomerase (dna) II-binding protein1 (TOPBP1), Cell division control protein 45homolog (CDC45), Mdm2- binding protein (MTBP), Minichromosome maintenance complex component 2 (MCM2), Protein MCM10 homolog (MCM10), Protein DBF4 homolog (DBF4), Serine/threonine-protein kinase Chk1 (CHEK1), DNA replication factor Cdt1 (CDT1), Cell division control protein 6 homolog (CDC6), and Bromodomain-containing protein 2 (BRD2) were recognized as the top ten correlated genes with TICRR in HCC. So far, studies have shown that TOPBP1 is a tumor suppressor gene which plays an inhibitory role in the progression of HCC. However, CDC45 and MCM2 play a promoting role in the progression and metastasis of HCC. In addition, GO enrichment analysis showed that TICRR co-expression is mainly related to “ATPase activity,” “tubulin binding,” “microtubule binding,” etc. KEGG analysis showed that TICRR co-expression is mainly related to “cell cycle” and “valine, leucine and isoleucine degradation.” These results may indicate that the expression of TICRR in HCC might play an important role in the tumorigenesis, progression, diagnosis, and predicting prognosis of HCC.

Aberrant methylation of DNA has been recognized as common epigenetic changes in human cancer (Zhu et al. 2018b). Therefore, DNA methylation is attracting more and more attention in the research of tumorigenesis and its early diagnosis and prognosis judgment (Sun et al. 2021; Zheng et al. 2020).

In recent years, clinical relationship between DNA methylation and tumor is becoming a hot focus of research. There is a great deal of research indicating that DNA methylation plays an important role in the occurrence and development of tumor, and DNA methylation is considered as one of the important critical mechanisms of tumorigenesis and progression in tumor (Horie et al. 2017; Tekpli et al. 2016; Zhang et al. 2019). In present study, high TICRR methylation levels of cg05841809, cg09403165, and cg03312532 were dramatically correlated with the prognostic values in HCC (*P* < 0.05). In addition, patients with high TICRR methylation of cg05841809, cg09403165, and cg03312532 CpG sites had a worse overall survival (OS) than patients with low TICRR methylation. The tumor microenvironment is composed of tumor cells, stromal cells, and the extracellular stroma. As an important part of the tumor microenvironment, the role of immune microenvironment is atracting more and more attention in the research of tumor immune microenvironment (Dhiman et al. 2021). Tumor immune microenvironment is not only the cause of tumor occurrence and development, but also the result of tumor tumorigenesis and progression (Petersson et al. 2022).

In this study, we found that the expression level of TICRR was noticeably positively correlated with infiltrating levels of B-cells, CD8 + T-cells, CD4 + T-cells, macrophages, neutrophils, and DCs in HCC. Moreover, we found that the expressions of PDCD1, CTLA4, LAG3, and TIGIT are positively correlated with TICRR in HCC. PDCD1, CTLA4, LAG3, and TIGIT are important markers in T-cells which can bind to tumor cell surface ligands to induce T-cell exhaustion. T-cell exhaustion is the key reason why the immune system cannot effectively eliminate chronic virus infection and malignant tumor. These results may indicate that TICRR probably promotes development and progression of HCC through the activation of immune molecules and immune cell biomarkers.

## Conclusions

In this study, we comprehensively analyzed the role of TICRR gene in HCC using The Cancer Genome Atlas (TCGA), Kaplan–Meier Plotter, TIMER (https://cistrome.shinyapps.io/timer/), GEO, STRING 11.0 (https://string-db.org), and various public databases. In conclusion, a better understanding of how the TICRR affects the development and progression of HCC would provide a new method for immune therapy of HCC. Therefore, TICRR might be used as a novel therapeutic target and prognostic biomarker for HCC gene therapy through regulating immune microenvironment.

### Supplementary Information

Below is the link to the electronic supplementary material.

Supplementary file1 (XLSX 39 kb)
